# An Evaluation of the Impact of Curdlan and Buttermilk Addition on the Functional Properties and Sensory Quality of Processed Cheese Analogs

**DOI:** 10.3390/molecules30010066

**Published:** 2024-12-27

**Authors:** Marika Magdalena Bielecka, Aleksandra Florczuk, Marek Aljewicz

**Affiliations:** Department of Dairy Science and Quality Management, Faculty of Food Science, University of Warmia and Mazury, Oczapowskiego 7, 10-719 Olsztyn, Poland; marika.bielecka@uwm.edu.pl (M.M.B.);

**Keywords:** designer foods, sensory evaluation, beta-glucan, texture, rheology, cheese microstructure

## Abstract

The present study was undertaken to investigate the impact of curdlan and buttermilk addition on the physicochemical and sensory attributes of processed cheeses (PCs), thereby elucidating their potential utility in culinary applications. Comprehensive analyses were conducted to assess the chemical composition, textural and rheological properties, microstructural features, and sensory characteristics of PCs. The findings indicate that the addition of curdlan notably decreased both the hardness and stickiness of cheeses but also significantly reduced their meltability. Conversely, an increase in buttermilk content was correlated with enhanced hardness of the cheese matrix. In addition, buttermilk provided a creamier taste, thereby increasing the product’s appeal for consumers. The colorimetric analysis revealed that buttermilk-induced cheese darkening via the Maillard reaction, whereas curdlan addition resulted in a modest increase in yellowness. Buttermilk-containing cheeses received high scores for smoothness and flavor in sensory evaluations. A marked decrease in the functional properties of PCs, such as meltability, was observed when buttermilk addition exceeded 2.5%. Overall, the results of this study suggest that the controlled addition of curdlan and buttermilk positively affects the texture, color, and flavor of PCs, and it provides valuable information for the cheese industry.

## 1. Introduction

Processed cheeses (PCs) are stable oil-in-water emulsions that contain dairy proteins, fat, emulsifying salts, and other ingredients. PCs are derived primarily from natural cheese, and their composition differs from cheese analogs that often contain non-dairy fats and proteins as substitutes [1]. The demand for reduced-fat products has greatly increased due to growing levels of consumer awareness of the relationship between diet and health [2]. PC analogs are products in which natural cheeses have been replaced with milk-based ingredients (caseinates or whey proteins) or non-dairy polymers (e.g., hydrocolloids, mainly polysaccharides, and selected proteins). Hydrocolloids contribute unique food functionalities due to their structural specificity, and they have been shown to be promising ingredients for attracting water, shaping and manipulating cheese structure, and modifying textural properties. Hydrocolloids play the role of active modifiers rather than inactive fillers in the cheese matrix. Similarly to fat, hydrocolloids can influence many sensory attributes of the matrix, such as hardness, cohesiveness, and spreadability [3]. Numerous studies have examined the applicability of hydrocolloids in the production of PC [4,5,6,7]. In recent years, the replacement of milk fat with β-glucans in dairy products has received considerable attention due to their potential health benefits and functional properties [4,8,9,10]. Curdlan is a bacterial polysaccharide classified as a β-glucan, composed of linearly oriented glucose units linked via β-(1→3)-glycosidic bonds. In aqueous solutions, curdlan forms thermoreversible gels upon heating, and it significantly influences the functional properties of food products [11]. Curdlan can promote the formation of a gel network in heated milk, thus contributing to the rheological behavior of the end product [12]. This attribute is particularly advantageous in low-fat cheese spreads and cheese dips with a continuous aqueous phase. Research indicates that the incorporation of curdlan into dairy products enhances their textural attributes, stability, and rheological profiles. Due to its unique gelling properties, curdlan can partially replace fat in food formulations while preserving the desired sensory characteristics of the final product [4,10]. In regards to the health-promoting properties of curdlan, its utilization can lower the caloric content of products without compromising their overall quality [12]. Milk proteins and their derivatives are extensively utilized in the production of PCs and their analogs. Buttermilk, a byproduct of butter manufacturing, contains water-soluble milk components, including casein, whey proteins, lactose, and milk fat globule membrane (MFGM) components, all of which exhibit excellent emulsifying properties [13]. Buttermilk is a good source of bioavailable calcium and magnesium. In terms of composition, sweet cream buttermilk (SCBM) closely resembles skim milk but is more abundant in phospholipids and MFGM proteins, which also possess strong emulsifying capabilities [14]. Due to its functional properties, buttermilk is increasingly used in the production of yogurts, functional beverages [15], ice cream [16], and various types of cheese [17,18,19,20], including cheese with reduced fat content [21]. The incorporation of buttermilk into cheese formulations can enhance product quality by improving the nutritional profiles, texture, viscosity, moisture retention, and sensory attributes of the resulting cheeses [17,19]. The use of buttermilk in dairy production aligns with sustainable development trends by promoting effective management of dairy byproducts. Additionally, the presence of bioactive compounds in buttermilk can augment the health-promoting properties of the final product, thus catering to the growing demand for functional foods.

The versatility of PCs stems from their specific physicochemical properties, such as meltability, stretching, and emulsion stability, which can be fine-tuned for various culinary applications. Meltability is a critical parameter that influences the functionality of cheese in applications that require smooth, homogeneous melting without phase separation, which is essential in sauces, dips, and toppings on pizzas and burgers. The rheological properties of PC, including its susceptibility to melting and stretching, are influenced by the content and proportions of water, proteins, and fats [22,23]. These properties are meticulously adjusted to meet specific application requirements and to ensure the functionality and sensory appeal of the final product. The manufacturing process and product composition are critical for standardizing the properties of PC [1].

Milk fat is replaced with other milk components to modify the chemical composition of the final product and enable the production of functional foods. The final acceptance of the product and, consequently, its marketability are determined by sensory attributes, such as color, texture, and palatability [24,25]. The use of curdlan, a gelling polysaccharide, in combination with buttermilk as a source of natural emulsifiers, can exert synergistic effects in the production of PC. Buttermilk, rich in whey proteins and phospholipids, contributes to the formation of stable emulsions and has a beneficial effect on the sensory properties of the final product. Consequently, the combination of curdlan and buttermilk can improve texture, consistency, and emulsion stability, as well as prolong the product’s shelf life.

However, the combined effect of curdlan and different concentrations of buttermilk on the functional properties of PCs has not been extensively studied to date. Therefore, the aim of the present study was to determine the effect of the addition of curdlan and different amounts of buttermilk on the most important functional properties of PC analogs.

## 2. Results and Discussion

### 2.1. Chemical Composition of Processed Cheese

PC analogs produced with the addition of curdlan and buttermilk were characterized by higher water content, which ranged from 64.49% to 66.86%, and accounted for 72.42–77.75% of moisture in the non-fat solids (MNFS) (Table 1). Compared to moisture content, MNFS had a more direct relationship with cheese properties. The addition of more than 2.5% buttermilk resulted in a significant decrease in fat content. The fat content of cheeses, expressed as fat in dry matter (FDM), was responsible for the desirable functional, textural, and sensory properties of cheese and ranged from 30.25 in cheeses with the highest buttermilk addition (DG 10) to 45.75 in cheeses with the addition of curdlan. The salt content of cheeses ranged from 1.24% to 1.37%, and salt in moisture (S/M) ranged from 3.63% to 3.93%. It is worth noting that S/M is strongly related to texture, rheology, and flavor [26]. The protein content of the tested cheeses ranged from 10.53% in PC analogs with the addition of curdlan (KDG) to 11.72% in control PC analogs (KK). The protein content of cheeses increased with a rise in the amount of added buttermilk (Table 1). The tested cheeses differed in pH, which was lowest (5.99) in PC analogs with the addition of curdlan and highest (6.29) in control PC analogs. However, in other experimental PC analogs, pH increased with a rise in the amount of added buttermilk due to their higher protein content.

### 2.2. Microstructure of Processed Cheese Analogs

**Figure 1:**
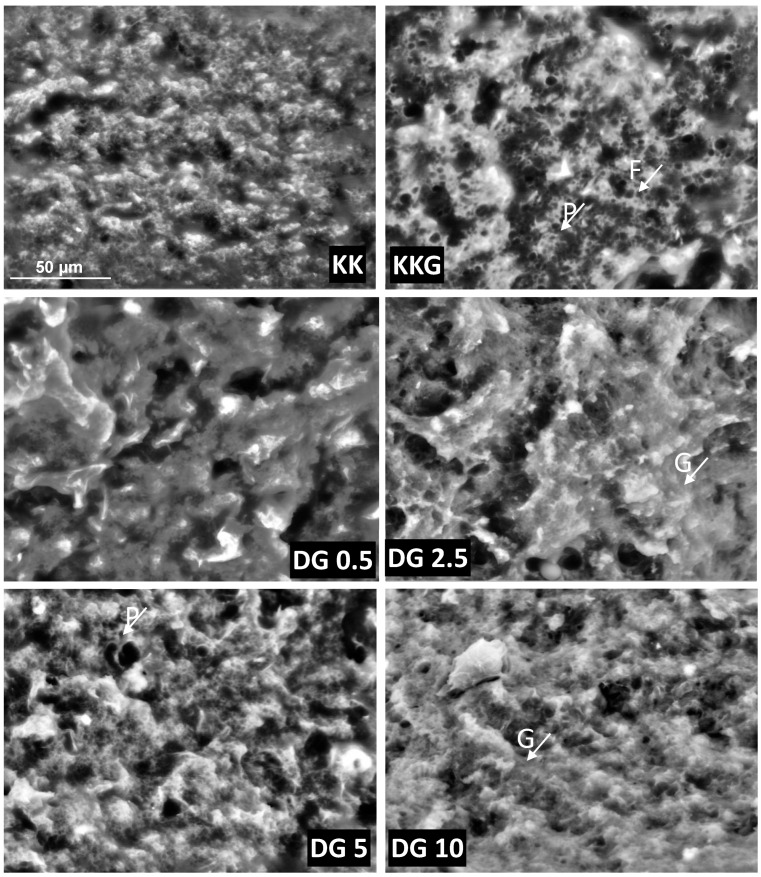
SEM images of PC analogs at 800× magnification. P—protein; F—fat; G—curdlan β-glucan. KK—control PC analogs; KDG—control PC analogs with curdlan; DG 0.5—PC analogs with curdlan and 0.5% buttermilk; DG 2.5—PC analogs with curdlan and 1% buttermilk; DG 5—PC analogs with curdlan and 5% buttermilk; DG 10—PC analogs with curdlan and 10% buttermilk.

The micrographs of control PC analogs, both without curdlan (KK) and with curdlan (KDG), revealed a compact, spongy, and irregular surface. In the control samples (KK), which contained neither buttermilk nor curdlan, the protein structure was typical for this type of product. It was uniform, with no visible clusters indicative of larger protein–protein complexes that are formed under the influence of temperature and shear forces (Figure 1). The fat globules in KK samples were homogeneously distributed, with an average size of 2.49 µm (in the range of 1.32 µm to 3.67 µm).

In KDG cheese analogs, the microstructure of the protein network was similar to that in KK samples. However, SEM images revealed cracks that were most likely caused by the addition of curdlan. By absorbing water, curdlan undergoes hydration and gellification. As a result, the protein–fat network is compressed, which simultaneously causes local stresses and cracks in the network [26]. These results are consistent with the findings of Konuklar et al. (2004), who studied the effect of the Nutrim fat replacer containing oat β-glucan on the microstructure of cheeses [27]. In contrast, Kratochvílová et al. (2022) did not observe cracks in the microstructure of the protein network of cheeses with 1% addition of carrageenan or agar, which suggests that the type of polysaccharide and product composition are crucial for microstructural stability [28]. Puff-like structures characteristic of polysaccharides were visible on the surface of PC analogs with the addition of curdlan. These structures occurred pointwise in the form of larger clusters, and similar observations were made in the authors’ previous study of PCs with the addition of curdlan and scleroglucan [4]. Unlike in the work of Florczuk et al. (2022), in the present study, polysaccharides did not cover the entire surface of cheese samples, which is not visible in the presented micrographs, and these discrepancies can be most likely attributed to differences in chemical composition and production technology [4]. A minor increase in the size of fat globules (around 3 µm on average, in the range of 1.59 µm to 4.81 µm) was noted in KDG cheese. Although the increase in average fat globule diameter was not significant, the above observation suggests that by interacting with proteins, curdlan contributed to the coalescence and redistribution of fat within the protein–fat network. These subtle changes could affect the textural characteristics and melting behavior of PC.

As a rich source of phospholipids and proteins, buttermilk acted as an effective emulsifier by forming a protective layer around fat globules. As a result, buttermilk addition prevented fat globules from coalescing and contributed to a uniform microstructure. In PC analogs with the lowest addition of buttermilk (DG 0.5), the diameter of fat globules decreased by approximately 14% to around 2.6 µm. In samples where buttermilk addition was increased to 10% (DG 10), fat globule diameter was reduced by 64% to 1.09 µm. These results suggest that fat was more effectively emulsified in DG cheeses than in cheeses with the addition of curdlan (KDG). These findings corroborate the results reported in a study by Kelimu et al. (2017), where buttermilk decreased particle size and promoted uniform particle distribution within the product’s microstructure [29]. It should also be noted that by inducing partial emulsification of casein and whey proteins, fat globule components in buttermilk could have affected the mutual distribution of proteins, fat, and curdlan within the microstructure.

### 2.3. Rheological Properties

**Figure 2:**
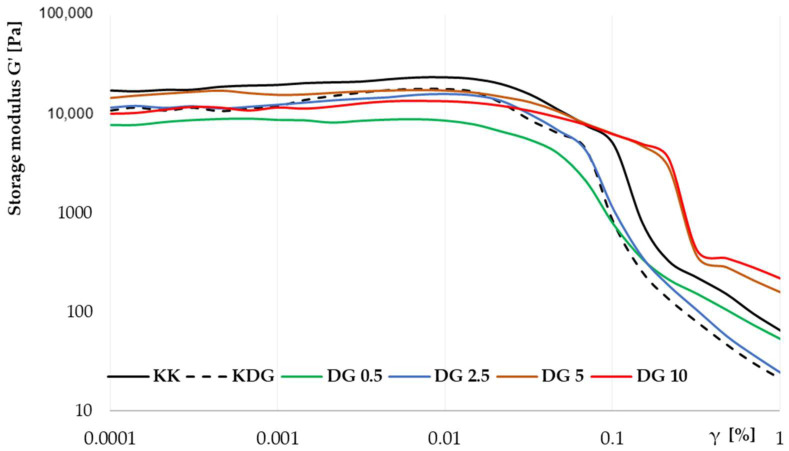
Amplitude sweep curves of the storage modulus (G′) of PC analogs. KK—control PC analogs; KDG—control PC analogs with curdlan; DG 0.5—PC analogs with curdlan and 0.5% buttermilk; DG 2.5—PC analogs with curdlan and 2.5% buttermilk; DG 5—PC analogs with curdlan and 5% buttermilk; DG 10—PC analogs with curdlan and 10% buttermilk.

A rheological analysis provided direct insights into the functional and mechanical properties of PC analogs. All melted cheeses exhibited shear-thinning pseudoplastic properties. Apparent viscosity values showed the highest (>99%) agreement with the power law model. The KK cheese was characterized by the smallest positive rheological exponent (*n* = 0.540) and a consistency coefficient (κ) of around 130, indicating that the product had relatively high viscosity and moderate pseudoplastic properties. These findings can be attributed to a well-structured spatial network of protein and fat, which was also confirmed in SEM images. In oscillatory measurements, the loss modulus G′ had the highest value (17,609 Pa), confirming the high elasticity of the analyzed product. In addition, KK cheese was characterized by the widest linear viscoelastic region (LVER) of around 3.2%, which further confirmed the high viscoelastic properties of the product (Figure 2).

One of the main reasons for using stabilizers such as curdlan is to improve the microstructure of products through synergistic interactions with proteins. In previous studies, 1% carrageenan was added to cheese [28], and 1–5% curdlan was added to whey protein gels [30]. In the present study, an analysis of cheeses with 0.5% curdlan addition revealed that even very low curdlan concentrations can induce significant changes in the rheological characteristics of a product. A significant decrease in the rheological index (p), approximately 80% decrease in apparent viscosity (η_app_), and the lowest κ value of only 50.4 were noted. Moreover, G′ values decreased by around 56% to 7800 Pa, without significant changes in the linearity range (LVER). The measurements conducted at variable amplitude showed that curdlan affected rheological properties, but not in a way that promoted the formation of a strong spatial protein network, as reported by Hu et al. (2024) [30]. These results also suggest that texture may be less stable in the long term, implying a decrease in functional properties. Despite being a neutral polysaccharide, curdlan had a significant effect on proteins. In the current study, at temperatures above 80°C, the structure of curdlan changed from a single helix to a triple helix, whereas proteins underwent conformational changes, such as the exposure of sulfhydryl groups and the formation of disulfide bonds that stabilize the gel structure. These changes are consistent with those observed in other studies where significant changes in protein structure occurred even at low curdlan concentrations, affecting the microstructure of cheese, although to a lesser extent than at higher concentrations [30]. The above findings only partially corroborate the results of the present study. Although an improvement in cheese microstructure was evident in SEM images, the key rheological parameters such as η_app_ and G′ clearly deteriorated. These observations indicate that more complex protein systems require further research to expand our understanding of the mechanisms by which curdlan affects the rheological properties and microstructure of cheese.

**Figure 3:**
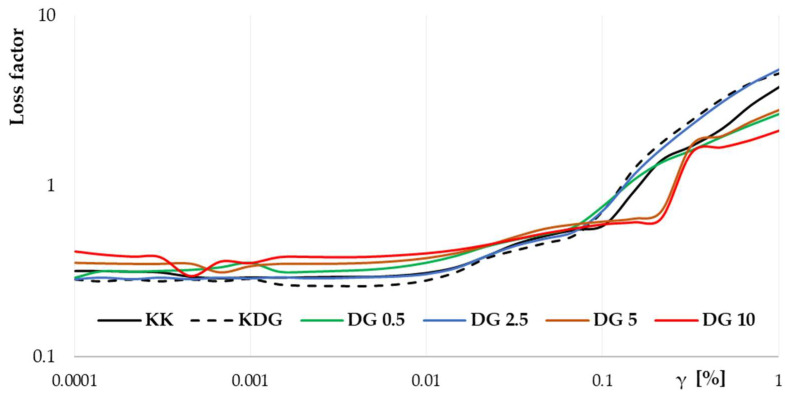
Loss factor curves of the tan δ = G″/G′ (tangent delta) of PC analogs. KK—control PC analogs; KDG—control PC analogs with curdlan; DG 0.5—PC analogs with curdlan and 0.5% buttermilk; DG 2.5—PC analogs with curdlan and 2.5% buttermilk; DG 5—PC analogs with curdlan and 5% buttermilk; DG 10—PC analogs with curdlan and 10% buttermilk.

The results of the statistical analysis show that the addition of buttermilk exerted a greater influence on the η_app_ of cheeses than curdlan, suggesting that buttermilk significantly affected the rheological properties of the analyzed products. In sample DG 0.5 with the lowest buttermilk addition, the elastic modulus G′ increased in the strain range of 0.09% to 2.16%, indicating an increase in the stiffness of the protein–curdlan–fat network. This observation could be attributed to the stabilizing effect of curdlan, which contributed to the formation of a stable network despite its low addition. However, the observed effect is not typical and depends on the type and dose of stabilizer [29]. DG 0.5 cheeses were also characterized by the most rapid decrease in η_app_ values with an increase in the shear rate, indicating that their structure was more susceptible to dilution, probably due to the interactions between curdlan and other components and optimized mutual distribution of particles, i.e., protein, fat, and lactose [31]. In contrast to DG 0.5, in cheeses with higher buttermilk content, the MFGM components released during production were bound to proteins and stabilized cheese microstructure. The above resulted in smoother product dilution with an increase in shear force [32]. Further analyses showed that the value of the consistency coefficient (κ) increased, whereas the rheological parameter *p* decreased with increasing buttermilk addition, indicating the thickening of the cheese microstructure due to an increase in protein and lactose content. Although the addition of more than 2.5% buttermilk improved the elasticity of cheeses at lower strain ranges, an increase in brittleness was observed at higher strain ranges, as evidenced by a sharp decrease in G′ at higher strains (Figure 2) and an increase in the loss ratio tan δ (Figure 3). The spatial structure of the protein network was destabilized when a certain limiting concentration of lactose was exceeded, leading to an increase in cheese brittleness. Similarly to curdlan, lactose competes with proteins for water in the system, which leads to protein dehydration, increases network rigidity, and decreases network elasticity.

### 2.4. Textural Properties

**Figure 4:**
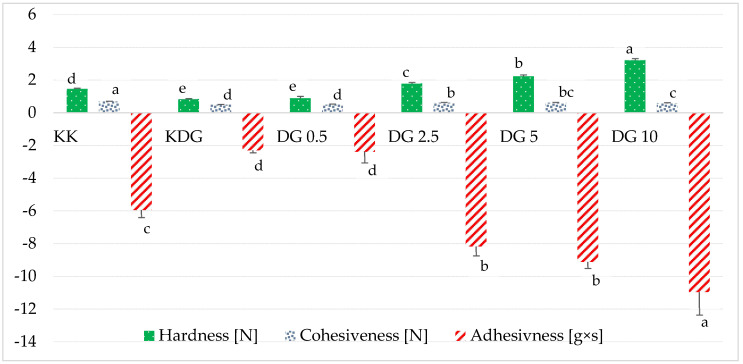
Textural properties of PC analogs. The values with the same color of columns with different letters (a–e) differed significantly at *p* ≤ 0.05. KK—control PC analogs; KDG—control PC analogs with curdlan; DG 0.5—PC analogs with curdlan and 0.5% buttermilk; DG 2.5—PC analogs with curdlan and 2.5% buttermilk; DG 5—PC analogs with curdlan and 5% buttermilk; DG 10—PC analogs with curdlan and 10% buttermilk.

The versatility of PCs could be attributed to their unique functional properties, which determine suitability for various culinary applications. In addition to appearance and aroma, texture was one of the sensory attributes that determined the acceptability and, consequently, the quality of PCs. The textural analysis of PCs revealed that differences in product composition and the additives used in the production process directly influenced their culinary use. The hardness of PC analogs was defined as the ability of a structured network to resist deformation when external force was applied, both at room temperature and low temperatures. Hardness also influenced the products’ sliceability and spreadability, which had important practical implications for implementing the analyzed technology. The addition of curdlan to PC analogs significantly decreased hardness from 1.47 N to 0.84 N (Figure 4), which was also consistent with the results of the rheological analyses. Florczuk et al. (2022) reported that the networks formed by protein and β-glucan were less developed than those formed by protein and fat [4]. The addition of polysaccharides contributed to a softer texture in different cheese matrices [33,34]. In the work of Solowiej et al. (2015), partial fat replacement with 1% inulin significantly decreased the hardness of PC analogs [35]. Da Silva et al. (2016) also noted that the addition of very small amounts of commercial konjac glucomannan (up to 0.25%) decreased the hardness of PC analogs, whereas larger amounts (0.5%, 0.75%, 1%, and 1.5%) increased hardness by increasing the demand for free water due to polysaccharide hydration [36]. The addition of 5% and 10% buttermilk significantly increased the hardness of the PC analogs (to 2.22 and 3.22 N, respectively) due to their lower fat content (Table 1) and a more compact and homogeneous microstructure (Figure 1). Fat acted as a filling phase in the protein matrix of PC analogs. By filling the spaces between proteins, fat reduced their mutual interactions, which decreased the rigidity of the protein structure. Therefore, cheeses with lower fat content had a less homogeneous and more compact microstructure, which increased the hardness of PC analogs. Similar observations were made by Zheng et al. (2016) [26] and Raval and Mistry (1999) [37], who found that a decrease in MNFS and FDM increased the hardness of sliced cheese. In addition, the increased hardness of PC analogs with the addition of buttermilk could also be attributed to the presence of intact casein (present in buttermilk powder), as confirmed by Purna et al. (2006) [38]. Intact casein intensified protein cross-linking, thus increasing resistance to mechanical deformation, i.e., product hardness. In summary, the hardness of PC analogs is determined by the complex interactions between proteins and fat. High-fat content moderated these interactions and contributed to a softer texture, while additives such as buttermilk powder, rich in intact casein, strengthened the protein network and increased product hardness.

The analysis revealed significant correlations between the textural properties and the chemical and sensory characteristics of PC analogs, where several associations were statistically significant at *p* ≤ 0.05 (Table 2). Hardness was bound by a strong positive correlation with adhesiveness (0.96) and work of shear (0.90), which indicates that harder cheeses tend to be more adhesive and require more energy to deform. In addition, hardness was positively correlated with mouthfeel characteristics such as texture (0.78) and viscosity (0.86), suggesting that a firmer texture enhances sensory experiences. Hardness was bound by a strong negative correlation with fat content (−0.94), indicating that high-fat cheeses were softer. Hardness was also negatively correlated with MNFS (−0.89) and fat in DM (−0.97), highlighting the impact of moisture and fat on texture.

The adhesiveness of different types of cheese is associated with the force needed to overcome the attraction between the cheese and the surface to which it adheres. In sensory analyses, adhesiveness denotes the degree to which a cheese sample sticks to the teeth during chewing. Negative values of adhesion indicate that the force acts downward to overcome the resistance and pull the cheese away from the surface. Higher negative values indicate that cheese is stickier during chewing, while lower values indicate that the cheese is less likely to adhere to teeth. Consumers tend to avoid sticky PCs/analogs that are difficult to separate from the packaging material. In the current study, the addition of curdlan reduced adhesiveness (Figure 4). Similar results were obtained by Solowiej et al. (2015), who noted that partial replacement of milk fat with 1% inulin significantly decreased adhesiveness, whereas greater substitution of fat with inulin increased the adhesiveness of PC analogs [35]. Adhesiveness, or cheese adhesion, was lowest in sample KDG (−2.3 ± 0.14 g × s), which suggests that this cheese was least viscous and most prone to peeling from the surface. In contrast, the highest adhesion work was noted in sample DG 10 (−10.97 ± 1.39 g × s), indicating that it was more viscous (Table 3). Adhesiveness showed a strong positive correlation with texture and mouthfeel, particularly with viscosity (0.76), implying that more adhesive cheeses have a thicker mouthfeel. The correlation with hardness (0.96*) also suggests that firmer cheeses exhibit greater adhesiveness. Adhesiveness was negatively correlated with fat content (−0.91) and MNFS (−0.81), which confirms the observation that adhesiveness increases with a decrease in fat content. Additionally, sodium chloride was positively correlated with adhesiveness (0.58), which indicates that sodium chloride can potentially influence this textural attribute (Table 3). In the work of Zheng et al. (2016), cheese adhesiveness was negatively correlated with protein content and positively correlated with FDM and S/M. Lower protein content and higher fat content were found to promote meltability and increase the adhesiveness of cheese [26].

It is interesting to note that despite the differences in hardness and viscosity, the cohesiveness of PC analogs remained fairly constant (in the range of 0.5–0.7), implying that all samples had similar microstructural homogeneity (Figure 4). In general, cohesiveness was bound by a weaker correlation with sensory attributes, but it was significantly correlated with MNFS (−0.37), indicating that cohesiveness was influenced by moisture content. Cohesiveness was positively correlated with protein content (0.62), indicating that higher protein content enhanced the cohesiveness of cheese (Table 2).

The work of shear (spreadability) was highest in sample DG 10 (1232.53 ± 48.18 g × s), which implies that this sample required the largest amount of energy to deform and, consequently, had the most cohesive structure and was least spreadable. The work of shear was lowest in the sample with curdlan (KDG, 255.82 ± 6.35 g × s), which confirms that this sample had the least compact structure (Table 3). Sample KDG was also characterized by the greatest spreadability. The work of shear was positively correlated with texture and mouthfeel attributes, including viscosity (0.86), suggesting that the ability to withstand shear forces was related to sensory characteristics. The work of shear was bound by a weaker correlation with MNFS (0.03), indicating that this parameter was less influential than other attributes. The presence of a strong negative correlation between the work of shear and fat in DM (−0.90) suggests that higher fat content may decrease the shear resistance of cheese (Table 2).

### 2.5. Meltability

In the analyzed groups of PC, meltability was highest in the control KK cheese (9.7 points). The meltability of the experimental cheeses was more influenced (*p* ≤ 0.05) by the amount of added buttermilk rather than the addition of curdlan alone (Table 3). Despite the fact that fat content was 0.58% higher and water content was 1% higher in KDG cheeses than in KK cheeses, the former were characterized by lower meltability due to the interactions between all product ingredients. According to Atik and Huppertz (2023), a higher content of fat and water generally improves cheese meltability due to the plasticizing effect of fat and less cross-linking of the microstructure [39]. However, in the present study, KDG cheeses were characterized by lower meltability, which could be attributed to the presence of curdlan, which limited water availability and mobility. In addition, curdlan formed a gel-like structure that coated protein–fat structures and further decreased their meltability. Similarly to curdlan, other hydrocolloids, such as starch [40], carrageenan [6], and Chios mastic gum [7], affected cheese meltability by interacting with proteins, influenced the water-holding capacity and, consequently, the microstructure of the analyzed products. Interestingly, although the low (0.5%) addition of buttermilk significantly reduced meltability to 9 points, the lubricity of this product was still higher than that of KDG. Most likely, the small amount of phospholipids and fat globule membrane components in samples with low buttermilk addition may have stabilized the fat–water emulsion phase and normalized fat globule size despite the presence of curdlan. These factors increased the homogeneity of the microstructure, which had a beneficial effect on cheese meltability.

However, it should be noted that increased buttermilk addition did not improve cheese meltability but rather decreased this parameter (Table 3). Increased buttermilk addition significantly decreased fat content, which, together with an increase in protein content, induced significant changes in the pseudo-melting characteristics of cheese (Table 1). These characteristics played a key role in the melting behavior of cheese. Importantly, despite the reduction in fat globule size, the protein–fat network was stabilized by curdlan, which reduced water mobility, and, in samples with higher buttermilk addition, also by lactose and protein. As a result, the structure of cheese became more rigid (as confirmed by the results of rheological and textural analyses), more compact (according to SEM results), and less susceptible to temperature changes. During melting, the protein network absorbed heat energy, which intensified the interactions between proteins and curdlan, stabilizing its structure [41]. In addition, the hydrophobic interactions between unfolded proteins and curdlan promoted further stiffening of the protein–curdlan–fat network, which decreased cheese meltability.

### 2.6. Color of Processed Cheese

The color of cheese significantly affects consumer acceptance because specific colors are associated with certain flavors. The color and appearance of cheese have been shown to influence consumer preferences and identification of cheeses, especially those with reduced fat content [42]. Cheese color not only attracts attention, but also provides cues about flavor and quality, which is why it is a crucial factor in sensory evaluations. In the present study, the color of cheese was assessed visually by a team of evaluators, and it was also measured with instrumental methods to avoid subjectivity. The results of the color analysis of PC samples are shown in Table 1. The average value of parameter L* (lightness) was approximately 85 in all samples. Compared to the control KK sample (86.49), the addition of curdlan did not induce significant changes in L* values, which suggests that curdlan did not exert a significant effect on the lightness of the samples in the experimental model (Table 1). Increasing amounts of added buttermilk led to a significant reduction in L* values, and sample DG 10 was characterized by the darkest color (L* = 83.05), probably due to lactose-induced Maillard reactions, such as the production of melanoidins [43], which also contributed to shifts in b* (yellow-blue) values with increasing buttermilk concentrations (Table 1). Higher b* values in the experimental PCs could be attributed to a higher content of hydroxymethylfurfural (HMF), which is formed when lactose reacts with free amino acids or proteins, and similar results were reported in studies examining Maillard reaction products [44]. The present results are also consistent with the findings of Kůrová et al. (2022), who investigated the effect of κ-carrageenan on the functional properties of melted cheese sauces [45]. It was also found that the value of parameter a* (red-green) was negative in all tested cheeses, indicating the predominance of green over red color. The addition of more than 0.5% buttermilk decreased the value of parameter a* and increased the contribution of greenness (Table 1). Similarly to b* and L* values, these changes were induced by biochemical transformations during the production of PC analogs, as well as by differences in the microstructure of the analyzed products, which reflected light differently, depending on the proportions of individual ingredients.

### 2.7. Sensory Properties of Processed Cheese

**Figure 5:**
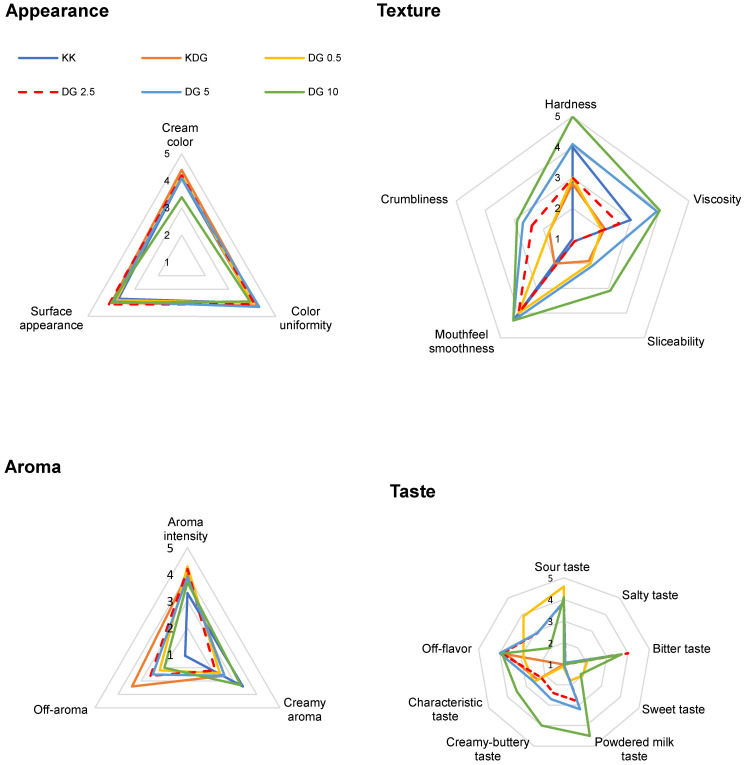
Sensory characteristics of PCs analogs. KK—control PC analogs; KDG—control PC analogs with curdlan; DG 0.5—PC analogs with curdlan and 0.5% buttermilk; DG 2.5—PC analogs with curdlan and 2.5% buttermilk; DG 5—PC analogs with curdlan and 5% buttermilk; DG 10—PC analogs with curdlan and 10% buttermilk.

All PC analogs had a uniform creamy color, and DG 10 cheeses were characterized by significantly lower intensity of the creamy color, suggesting that higher concentrations of buttermilk significantly contributed to a decrease in the creamy color score. The addition of buttermilk and curdlan had no effect on the appearance of cheese surfaces. In contrast, PC analogs differed in texture parameters assessed by the panelists in the sensory evaluation. Cheeses with 10% buttermilk addition were hardest, whereas cheeses with curdlan addition (KDG) and those with 0.5% and 2.5% buttermilk addition (DG 0.5 and DG 2.5, respectively) were softest. Cheeses with the addition of curdlan (KDG) were characterized by the lowest viscosity as well as the least smooth mouthfeel; therefore, the addition of curdlan without buttermilk reduced viscosity, while the addition of 5% and 10% buttermilk increased the viscosity of PC analogs. The examined PCs with the highest buttermilk addition (10%) were the most viscous (DG 10). Increased buttermilk addition to PC analogs resulted in a flourier texture. Regarding the sensation of fullness, the highest values were observed in the control cheeses and in cheeses with added curdlan (Figure 5).

However, palatability, i.e., taste and aroma sensation, was the most important sensory attribute of PC analogs. Palatability is the sensation activated when at least a dozen tastants and odorants present in cheese are recognized by taste and smell receptors. An increase in buttermilk content (up to 10%) enhanced saltiness and sweetness and led to the emergence of a distinct milk powder flavor and a creamy-buttery aftertaste. Buttermilk, rich in lactose and mineral salts, intensified the flavor of PCs, probably because these components contributed to the overall taste profile. In contrast, the addition of curdlan alone increased the intensity of the bitter taste in PC (KDG) and introduced a foreign flavor. In regards to aroma, cheeses with added glucan exhibited higher aroma intensity than the control cheeses (Figure 5). These findings suggest that buttermilk enhances desirable flavors, whereas curdlan may alter taste and aroma in ways that could affect consumer acceptance.

## 3. Materials and Methods

### 3.1. Materials

The raw materials for the study included six-week matured cheeses—Maasdam manufactured by Spółdzielnia Mleczarska w Rykach (Ryki, Poland) and Gouda manufactured by SM Mlekovita (Wysokie Mazowieckie, Poland). One-week low-fat quark supplied by OSM Łowicz (Łowicz, Poland) was also used in the study. The remaining dairy ingredients, including butter, milk powder, and buttermilk powder, were obtained from SM Mlekovita (Wysokie Mazowieckie, Poland). Whey protein concentrate was supplied by Polmlek (Lidzbark Warmiński, Poland).

Curdlan, a β-glucan derived from *Agrobacterium* spp., cacodylic acid, and glutaraldehyde, was purchased from Sigma-Aldrich (St. Louis, MO, USA). Sodium chloride, ethanol, and sodium citrate were obtained from Chempur (Piekary Śląskie, Poland).

### 3.2. Production of Processed Cheese Analogs

**Figure 6:**
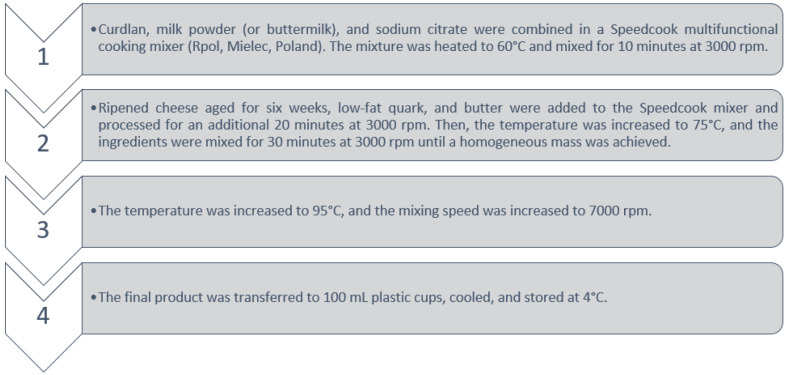
Production stages.

The proportions of the ingredients are shown in Table 4 and Table 5. The ratio of Gouda to Maasdam cheese was 1:1 *w*/*w*. The composition was normalized to achieve a ripened cheese-to-quark ratio of 80:20. The normalization process ensured a dry matter (DM) content of 32 ± 0.5% and a protein content of 10–11%. Cheese production was carried out in four stages, which are described in Figure 6.

### 3.3. Chemical Composition Analysis

Protein content was determined using the Kjeldahl method [46]; fat content was determined using the gravimetric method [47]; salt content was determined according to ISO 5943:2007 [48]. DM content was determined with a weigh-dryer according to the manufacturer’s instructions (Mettler-Toledo, Columbus, OH, USA). Fat in dry matter (FDM, g fat/100 g DM), moisture in non-fat solids (MNFS, g water/100 g non-fat solids), and salt in moisture (S/M) were calculated.

### 3.4. pH Analysis

The pH measurements were performed at room temperature using a glass-tip electrode connected to a pH meter (pH Elmetron, Zabrze, Poland). The electrode was inserted directly into the PC samples at six randomly chosen locations within each package.

### 3.5. Microstructure Analysis

The microstructure of PC samples was analyzed using a Quanta 200 SEM (FEI, Brno, Czech Republic) equipped with a dual BSD detector, as described by Florczuk et al. [4]. Briefly, cube-shaped samples (3 mm × 3 mm × 3 mm) were cut from a cheese block and fixed by immersion in 2.5% glutaraldehyde solution (Merck, Darmstadt, Germany) in 0.05 mol/L of cacodylic acid buffer (pH 7.2) for 24 h at 4°C. The samples were then dehydrated with a graded ethanol series (60%, 70%, 80%, 95%, and 99.8% (*v*/*v*)), each for 30 min. They were critical-point dried using a Leica EM CPD030 critical-point dryer (Leica Microsystems, Vienna, Austria). Microscopic analysis was carried out at a constant pressure of 130 Pa, accelerating voltage of 30 kV, and 800× magnification.

### 3.6. Rheological Properties

The rheological properties of PCs were evaluated using an MCR 102 rheometer (Anton Paar, Graz, Austria) equipped with temperature control units, including fluid circulators and a Peltier hood, according to the methodology described by Aljewicz et al. (2021) with some modifications [10]. A parallel plate geometry (PP25/S-SN73131) was used in the measurements. Data were acquired with RheoCompass software v. 1.31 (Anton Paar). At the outset, a normal force of 1 N was applied to ensure adequate contact between the sample and the plates, followed by a 3 min rest period to allow any residual stresses to dissipate. During the measurement, the normal force was kept constant at 0.4 N, and the gap was set at 1 mm. The linear viscoelastic region (LVR) was determined at 25°C by conducting a strain sweep from 0.001% to 100% at constant angular frequency of 1 Hz, using a logarithmic ramp with six measurement points per decade. The flow behavior of cheese samples was analyzed using a controlled shear rate (CSR) protocol over a shear rate range of 1–100 s^−1^ at 25 ± 1°C. To ensure accurate results, a recovery and temperature stabilization period of 180 s was allowed before each measurement.

### 3.7. Texture Analysis

The penetration test was conducted using a TA-XT2i texture analyzer (Stable Micro Systems, Surrey, UK) equipped with a stainless steel cylinder probe (P10, 10 mm diameter). The test was performed based on the protocol provided by the manufacturer. PC samples were prepared by pouring cheese into cylindrical cups with a diameter of 60 mm and a height of 70 mm. The samples were stored at 4 °C for at least 48 h to ensure uniform consistency. Prior to testing, the samples were equilibrated to room temperature (approximately 20–22 °C) by removing them from refrigeration 30 min before the test.

During the test, each sample was placed centrally on the texture analyzer platform. The cylinder probe was aligned with the center of the sample’s surface and then lowered into the sample at a constant speed of 1 mm/s until a depth of 30 mm was reached. The force required to penetrate the sample was recorded continuously using the Exponent software (version 6.2). Cheese samples were evaluated for hardness, adhesiveness, and cohesiveness. Six measurements were performed for each sample to ensure the accuracy and reproducibility of the results.

### 3.8. Spreadability Test

The spreadability of PC was evaluated with the TA-XT2i texture analyzer (Stable Micro Systems) equipped with a TTC spreadability rig (HDP/SR) and a 5/25 kg load cell (Stable Micro Systems). Shear work is the total force required for the spreading process, where lower values indicate that less force is needed to achieve spreadability. The samples were placed in a female cone (90° angle), and special attention was paid during the process to avoid bubble formation. The compression distance was set at 23 mm; test speed was 3 mm/s; post-test speed was 10 mm/s. The samples were analyzed at a temperature of 20 ± 1 °C immediately after being removed from the refrigerator (4 °C). All measurements were performed in a minimum of six replicates.

### 3.9. Meltability Test

The meltability test was performed according to the method described by Florczuk et al. [4]. Briefly, the meltability of the PC analog was measured using a modified Schreiber test. The samples (4.8 mm thick and 41 mm in diameter) were placed in Petri dishes and heated in a microwave oven (Speedcook, Mielec, Poland) at 300 W for 60 s and then removed and allowed to cool. The results of the Schreiber meltability test, measured on an arbitrary scale of 0 to 10 units, were expressed as the average of 3 measurements for each of the 3 replicates. Values below 4 were indicative of poor meltability; values between 4 and 7 were indicative of good meltability; values above 7 were indicative of excellent meltability.

### 3.10. Instrumental Color Analysis

The color of cheeses and cheese-like products was analyzed with a CM-3500d spectrophotometer (Konica Minolta Sensing, Inc., Osaka, Japan) that measures color transmittance and reflection. Measurements were performed with d/8 geometry, 8 mm aperture size, 10° observer angle, and D65 illuminant. Before analysis, the device was calibrated against a white calibration plate (CM A120) and a black calibration plate (CM A124). The color of PC products was evaluated after production. A representative sample was prepared immediately before analysis at room temperature and placed in a CM A-128 Petri dish (h = 25 mm, Ø = 34 mm). Color measurements were performed in a minimum of six replicates. Color lightness was calculated in the CIE LAB color space and expressed by the L* component, a coordinate corresponding to lightness (L* = 0 for black and L* = 100 for white). The chromaticity of the light source was described with components a* (−a* = greenness and +a* = redness) and b* (−b* = blueness and +b* = yellowness).

### 3.11. Sensory Analysis

The sensory analysis of PC was conducted in a sensory laboratory using the profiling method described in ISO 13299:2016-05E [49]. The analysis involved 8 panelists (4 women and 4 men, aged 22–50 years) who gave informed consent to participate in the study and signed the required consent forms. The panelists were trained in the sensory evaluation of dairy products according to ISO 8586:2014 [50] and validated for sensory sensitivity. The panelists assessed the intensity of 20 selected sensory attributes grouped into 4 categories: appearance, consistency, mouthfeel texture, taste, and aroma. The categories of the evaluated attributes included appearance (creamy color, color uniformity, and surface appearance), consistency (hardness, stickiness, and sliceability), mouthfeel texture (smoothness in the mouth, graininess, and full-fat perception), taste (sour taste, salty taste, bitter taste, sweet taste, powdered milk taste, buttery-creamy taste, typical taste, and off-flavor), and aroma (aroma intensity, creamy aroma, and off-aroma). The evaluation was conducted on a 5-point descriptive scale, where 1 denoted the absence of a given attribute and 5 denoted very high intensity of the tested attribute. Cheese samples of approximately 20 g each were coded with 3-digit numbers and served chilled at 7 °C in plastic cups. Each panelist was provided with a teaspoon for tasting, as well as crackers and a knife for assessing spreadability. The analysis took place on the third day after production, and the panelists used definition cards and score sheets to standardize the assessment. The tests were conducted in accordance with ISO 8589:2007 [51] to ensure the appropriate experimental conditions.

### 3.12. Statistical Analysis

The results were processed statistically by calculating the mean and standard deviation. Differences were determined by factorial analysis of variance (ANOVA) at a significant level of *p* ≤ 0.05. The Tukey test was used for post-hoc analysis. The presence of significant correlations between textural properties, sensory mouthfeel characteristics, and chemical composition was determined using Spearman’s rank correlation coefficient. All analyses were performed using Statistica 13.5 PL software (StatSoft, 2017, Krakow, Poland).

## 4. Conclusions

In summary, the research objective of this study was to evaluate the impact of curdlan and different amounts of buttermilk on the important functional properties of PC analogs. Buttermilk had a greater impact on the rheological characteristics of PC analogs, including texture, and then curdlan. Curdlan lowered the pH of the cheeses and reduced their meltability. Curdlan acted as a stabilizer and promoted the formation of a stable protein network. Buttermilk reduced the diameter of fat globules and stabilized the microstructure of the final product, which can be largely attributed to its unique composition, in particular the presence of fat globule membrane proteins and phospholipids. Buttermilk was more effective than curdlan in improving fat emulsification and contributed to a decrease in fat globule diameter. Curdlan reduced hardness, while buttermilk increased the hardness of PC analogs. Cheese adhesiveness was influenced by fat and protein content: viscosity increased with a rise in fat content and decreased with a rise in protein content. A significant deterioration in the functional characteristics of PCs was observed when buttermilk addition exceeded 2.5%. This was manifested by increased cross-linking of the microstructure, increased hardness, decreased elasticity, and lower lubricity of the final products. The color analysis and the sensory evaluation revealed that the addition of buttermilk, especially in higher amounts, resulted in a darker, creamy-buttery color of cheese and increased flavor intensity and sweetness. From a sensory perspective, samples with a higher proportion of buttermilk were characterized by increased firmness and a distinct flavor. 

The study demonstrated that both curdlan and buttermilk—normally a byproduct of dairy production—can be successfully used in the development of new, innovative dairy product formulations. Buttermilk improved emulsification and stabilized the microstructure, while curdlan modified the textural properties, collectively enhancing product quality and addressing the growing consumer demand for functional foods.

## Figures and Tables

**Table 1:** Composition, pH, and color attributes for PC analogs.

Composition	Abbreviation	KK	KDG	DG 0.5	DG 2.5	DG 5	DG 10
*w*/*w*%							
Moisture		64.49 ^d^ ± 0.18	65.47 ^ab^ ± 0.11	65.86 ^ac^ ± 0.27	65.77 ^ac^ ± 0.01	65.47 ^ab^ ± 0.81	64.69 ^d^ ± 0.17
Fat		15.22 ^b^ ± 0.27	15.80 ^b^ ± 0.08	15.26 ^b^ ± 0.04	14.23 ^a^ ± 0.24	12.69 ^e^ ± 0.86	10.68 ^c^ ± 0.15
Salt		1.31 ^ac^ ± 0.05	1.26 ^d^ ± 0.04	1.24 ^d^ ± 0.02	1.36 ^ab^ ± 0.02	1.37 ^b^ ± 0.01	1.33 ^abc^ ± 0.03
Dry matter	DM	35.52 ^d^ ± 0.18	34.53 ^ac^ ± 0.11	34.14 ^ab^ ± 0.27	34.24 ^ab^ ± 0.01	34.53 ^ac^ ± 0.81	35.31 ^d^ ± 0.17
Protein		11.72 ^e^ ± 0.06	10.53 ^b^ ± 0.04	10.76 ^bc^ ± 0.06	11.04 ^d^ ± 0.04	11.31 ^a^ ± 0.45	11.37 ^a^ ± 0.12
Moisture in non-fat solids	MNFS	76.06 ^e^ ± 0.06	77.75 ^a^ ± 0.12	77.72 ^a^ ± 0.30	76.68 ^f^ ± 0.22	74.98 ^d^ ± 0.50	72.42 ^b^ ± 0.14
Fat in dry matter	FDM	42.86 ^b^ ± 0.53	45.75 ^c^ ± 0.21	44.71 ^c^ ± 0.31	41.57 ^a^ ± 0.70	36.71 ^f^ ± 1.82	30.25 ^d^ ± 0.35
Salt in moisture	S/M	3.70 ^bc^ ± 0.16	3.64 ^bc^ ± 0.11	3.63 ^b^ ± 0.05	3.96 ^a^ ± 0.07	3.97 ^a^ ± 0.08	3.76 ^cd^ ± 0.07
Acidity	pH	6.29 ^a^ ± 0.01	5.99 ^d^ ± 0.01	6.00 ^d^ ± 0.00	6.22 ^c^ ± 0.01	6.26 ^b^ ± 0.01	6.28 ^a^ ± 0.00
Color	L*	86.49 ^a^ ± 0.18	86.30 ^a^ ± 0.07	86.16 ^a^ ± 0.20	85.24 ^d^ ± 0.21	84.58 ^c^ ± 0.37	83.05 ^b^ ± 0.68
	a*	−2.29 ^d^ ± 0.04	−1.87 ^a^ ± 0.04	−1.77 ^e^ ± 0.02	−1.93 ^a^ ± 0.07	−2.9 ^c^ ± 0.12	−3.36 ^b^ ± 0.06
	b*	12.30 ^d^ ± 0.09	14.00 ^a^ ± 0.09	14.11 ^a^ ± 0.10	14.14 ^ab^ ± 0.07	13.82 ^bc^ ± 0.23	14.87 ^e^ ± 0.36

All results had been presented as arithmetic mean and standard deviation calculated from 10 measurements. Data were analyzed using test ANOVA—post-hoc—Turkey test. a–f—mean values in rows marked with different letters differ significantly at *p* ≤ 0.05. KK—control PC spreads; KDG—control PC spreads with curdlan; DG 0.5—PC spreads with curdlan and 0.5% buttermilk; DG 2.5—PC spreads with curdlan and 2.5% buttermilk; DG 5—PC spreads with curdlan and 5% buttermilk; DG 10—PC spreads with curdlan and 10% buttermilk. L*, a*, and b* values for light to dark, red to green, and yellow to blue, respectively.

**Table 2:** Spearman’s rank correlation coefficients for textural and mouthfeel characteristics related to chemical composition.

	Hardness [N]	Adhesiveness [N × s]	Cohesiveness [N]	Work of Shear [g × s]
Adhesiveness [N × s]	0.96 *			
Cohesiveness	0.22	0.14		
Work of Shear [g × s]	0.90 *	0.88 *	0.10	
Texture mouthfeel—hardness	0.78 *	0.68 *	0.45 *	0.64 *
Texture mouthfeel—viscosity	0.86 *	0.76 *	0.44 *	0.86 *
Texture mouthfeel—sliceability	0.44 *	0.44 *	0.21	0.67 *
Moisture [*w*/*w*%]	0.05	0.16	−0.08	−0.12
Fat [*w*/*w*%]	−0.16	−0.12	−0.37 *	0.03 *
Chloride sodium [*w*/*w*%]	−0.94 *	−0.91 *	−0.24	−0.94 *
Total solid [*w*/*w*%]	0.58 *	0.56 *	−0.23	0.48 *
Protein [*w*/*w*%]	0.62 *	0.59 *	0.21	0.42 *
Moisture in non-fat solids	−0.89 *	−0.81 *	−0.32	−0.75 *
Fat in dry matter	−0.97 *	−0.92 *	−0.25	−0.90 *
Salt in moisture	0.55 *	0.54 *	−0.33	0.49 *

* Correlation coefficients are significant at *p* ≤ 0.05.

**Table 3:** Textural and rheological properties of PCs analogs.

	KK	KDG	DG 0.5	DG 2.5	DG 5	DG 10
Spreadability						
Work of Shear [g × s]	549.79 ^b^ ± 25.66	255.82 ^a^ ± 6.35	664.73 ^c^ ± 6.09	837.30 ^d^ ± 49.13	971.70 ^e^ ± 40.95	1232.53 ^f^ ± 48.18
Meltability						
	9.7 ^a^ ± 0.1	8.0 ^d^ ± 0.2	9.021 ^b^ ± 0.09	7.940 ^d^ ± 0.05	8.0 ^d^ ± 0.1	7.0 ^c^ ± 0.2
Rheology properties						
κ	129.64 ^cd^ ± 4.81	50.41 ^e^ ± 0.45	77.88 ^d^ ± 2.05	18.20 ^b^ ± 5.78	197.43 ^b^ ± 15.26	290.72 ^a^ ± 7.43
P	0.54 ^d^ ± 0.00	0.62 ^c^ ± 0.03	0.73 ^b^ ± 0.01	0.65 ^c^ ± 0.04	0.62 ^c^ ± 0.04	0.93 ^a^ ± 0.07
Apparent viscosity	476.92 ^f^ ± 8.40	97.39 ^e^ ± 4.76	500.03 ^d^ ± 1.93	637.00 ^c^ ± 2.65	738.59 ^b^ ± 3.06	1722.56 ^a^ ± 14.52
[Pa·s] at 0.1 [1/s]						

The results were expressed as arithmetic mean value and standard deviation from 10 measurements. Mean values in rows marked with different letters (a–f) differed significantly at *p* ≤ 0.05. Data were analyzed using one-way ANOVA followed by Tukey’s post-hoc test. KK—control PC analogs; KDG—control PC analogs with curdlan; DG 0.5—PC analogs with curdlan and 0.5% buttermilk; DG 2.5—PC analogs with curdlan and 2.5% buttermilk; DG 5—PC analogs with curdlan and 5% buttermilk; DG 10—PC analogs with curdlan and 10% buttermilk.

**Table 4:** Chemical composition [%] of the ingredients used in the study.

	Cheese Ripening	Quark	Butter	Milk	WPC	Buttermilk	Milk Powder
Fat	27.0	0.3	82.0	2.0	4.0	5.0	1.3
Protein	26.0	16.0	1.0	3.0	60.3	31.0	34.0
Salt	1.1	0.1	0.0	0.1	0.8	1.1	1.1
Carbohydrate	2.0	4.5	1.0	4.7	25.6	50.0	51.0

**Table 5:** Percentage content [w/w] of each ingredient in the analyzed products.

	KK	KDG	DG 0.5	DG 2.5	DG 5	DG 10
Gouda cheese	28.00	27.60	27.20	28.80	25.20	20.40
Quark	7.00	6.90	6.80	7.20	6.30	5.10
Butter	8.00	8.00	7.50	6.25	6.00	4.00
Milk	53.45	53.95	55.45	53.20	55.45	58.45
Buttermilk	0.00	0.00	0.50	2.50	5.00	10.00
Milk powder	2.00	1.00	0.00	0.00	0.00	0.00
WPC	0.00	0.50	0.50	0.00	0.00	0.00
Emulsifying salts	1.00	1.00	1.00	1.00	1.00	1.00
Curdlan	0.00	0.50	0.50	0.50	0.50	0.50
Salt	0.55	0.55	0.55	0.55	0.55	0.55

KK—control PC analogs; KDG—control PC analogs with curdlan; DG 0.5—PC analogs with curdlan and 0.5% buttermilk; DG 2.5—PC analogs with curdlan and 2.5% buttermilk; DG 5—PC analogs with curdlan and 5% buttermilk; DG 10—PC analogs with curdlan and 10% buttermilk.

## Data Availability

The results are available from the authors upon request.
